# Extension of the *in vivo* half-life of endostatin and its improved anti-tumor activities upon fusion to a humanized antibody against tumor-associated glycoprotein 72 in a mouse model of human colorectal carcinoma

**DOI:** 10.18632/oncotarget.3121

**Published:** 2015-01-22

**Authors:** Sang-Hyun Lee, In Cheul Jeung, Tae Woo Park, Kyungmin Lee, Dong Gwang Lee, Young-Lai Cho, Tae Sup Lee, Hee-Jun Na, Young-Jun Park, Hee Gu Lee, Mun Sik Jeong, Kwang-Hee Bae, Sang Chul Lee, Hyo Jin Lee, Young-Guen Kwon, Hyo Jeong Hong, Jang-Seong Kim, Jeong-Ki Min

**Affiliations:** ^1^ Functional Genomics Research Center, Korea Research Institute of Bioscience and Biotechnology, Daejeon, Republic of Korea; ^2^ Department of Obstetrics and Gynecology, College of Medicine, The Catholic University of Korea, Seoul, Republic of Korea; ^3^ Department of Biomolecular Science, University of Science & Technology, Daejeon, Republic of Korea; ^4^ Department of Chemistry, Dongguk University, Seoul, Republic of Korea; ^5^ Molecular Imaging Research Center, Korea Institute of Radiological and Medical Sciences, Seoul, Republic of Korea; ^6^ Scripps Korea Antibody Institute, Chuncheon, Republic of Korea; ^7^ Department of Systems Immunology, College of Biomedical Science and Institute of Antibody Research, Kangwon National University, Chuncheon, Republic of Korea; ^8^ Department of Internal Medicine and Cancer Research Institute, Chungnam National University School of Medicine, Daejeon, Republic of Korea; ^9^ Department of Biochemistry, College of Life Science and Biotechnology, Yonsei University, Seoul, Republic of Korea

**Keywords:** Angiogenesis inhibitors, Colorectal carcinoma, Endostatin, Tumor-associated glycoprotein-72, Vascular Endothelial Growth Factor

## Abstract

Endostatin is an endogenous angiogenesis inhibitor that exhibits potential anti-tumor efficacy in various preclinical animal models. However, its relatively short *in vivo* half-life and the long-term, frequent administration of high doses limit its widespread clinical use. In this study, we evaluated whether a fusion protein of murine endostatin (mEndo) to a humanized antibody against tumor-associated glycoprotein 72 (TAG-72), which is highly expressed in several human tumor tissues including colon cancer, can extend the serum half-life and improve the anti-tumor efficacy of endostatin by targeted delivery to the tumor mass. The fusion protein (3E8-mEndo) and mEndo showed improved anti-angiogenic activity *in vitro* and *in vivo*, predominantly by interfering with pro-angiogenic signaling triggered by vascular endothelial growth factor (VEGF). Moreover, in mice treated with 3E8-mEndo, we observed a markedly prolonged serum half-life and significantly inhibited tumor growth. The improved anti-tumor activity of 3E8-mEndo can be partially explained by increased local concentration in the tumor mass due to targeted delivery of 3E8-mEndo to implanted colon tumors. Collectively, our data clearly indicate that tumor-targeting antibody fusions to endostatin are a powerful strategy that improves the poor pharmacokinetic profile and anti-tumor efficacy of endostatin.

## INTRODUCTION

Angiogenesis, the formation of new blood capillaries from preexisting vessels, plays a key role in tumor growth and metastasis. Therefore, angiogenesis is considered an attractive target for anticancer therapies. Currently, several angiogenesis inhibitors are approved for the treatment of cancer [1]. Because these inhibitors target only rapidly proliferating tumor-associated endothelial cells and not relatively dormant endothelial cells in normal tissue, anti-angiogenic therapies are expected to be less toxic than conventional chemotherapy. Moreover, targeting relatively homogenous and genetically stable cells, such as endothelial cells, may avoid acquired drug resistance.

Endostatin, a 20 kDa internal fragment of the C-terminus of collagen XVIII, is an endogenous inhibitor of angiogenesis. It has strong *in vitro* and *in vivo* anti-angiogenic activities, and exhibits potential anti-tumor efficacy in a variety of preclinical animal models of human carcinomas, including metastatic colorectal cancer [2]. Although the mechanism of endostatin action is not fully understood, endostatin appears to exert its anti-angiogenic properties via interaction with several cell-surface receptors on endothelial cells. For instance, endostatin directly binds to VEGFR-2 (vascular endothelial growth factor receptor-2; KDR/Flk-1), which subsequently inhibits vascular endothelial growth factor (VEGF)-induced ERK and p38 mitogen-activated protein kinase (MAPK) activation [3]. Endostatin suppresses endothelial cell migration by binding integrin α_5_β_1_ and thus inhibiting focal adhesion kinase (FAK) activation [4]. In addition, endostatin has been reported to inhibit Wnt-signaling [5], and to induce cell cycle arrest in endothelial cells by inhibiting cyclin D1 [6]. Despite these promising anti-angiogenic and anti-tumor activities in preclinical settings, endostatin clinical trials did not demonstrate significant therapeutic benefits for patients with cancer.

Because anti-angiogenic therapy is cytostatic rather than cytocidal, chronic administration of relatively high doses of the therapeutic proteins is required to maintain consistent and effective protein concentrations, thus ensuring tumor growth suppression. Moreover, many recombinant therapeutics, including endostatin, have a relatively short *in vivo* half-life. Therefore, novel strategies that enhance the *in vivo* half-life and improve patient compliance are urgently required to enable the widespread clinical use of these proteins.

Several strategies have been reported to increase the *in vivo* half-life of therapeutic proteins. Among them, the most widely used and successfully adopted strategy is fusion of the Fc-region of immunoglobulin G or the whole antibody to the therapeutic protein [7]. Moreover, targeted delivery strategies have also been developed to increase the local, rather than systemic, concentration of therapeutic proteins in the tumor by fusing the proteins to antibodies against tumor-specific antigens [8, 9].

Tumor-associated glycoprotein (TAG)-72 is overexpressed in many carcinoma tissues, including colorectal, ovarian, breast, gastric, and pancreatic, compared with corresponding normal tissues [10, 11]. Previously, we reported that a mutant version of the humanized antibody against TAG-72, designated a 3E8, has a higher binding affinity, but negligible immunogenicity, compared with the original murine monoclonal antibody (CC49) or humanized antibody (huCC49). Moreover, 3E8 was efficiently delivered to the tumor and persistently localized in the tumor mass. The plasma stability of 3E8 was highly increased compared to the huCC49 antibody [12]. These properties suggest that 3E8 may be a potential carrier to deliver therapeutic proteins to tumors expressing TAG-72, such as colorectal carcinoma, thereby increasing the local concentration of therapeutic proteins.

This study was conducted to evaluate whether fusion of endostatin to the C-terminus of the 3E8 antibody can improve the *in vivo* half-life and anti-tumor efficacy of endostatin, thus improving its clinical applicability. Because this fusion protein significantly improved the pharmacokinetic behavior and anti-tumor efficacy of endostatin via selective delivery and enhanced retention in tumor masses expressing TAG-72 protein without adversely affecting its anti-angiogenic activities, fusion of 3E8 to endostatin could be a useful therapeutic strategy

## RESULTS

### Expression and purification of anti-3E8-mEndo fusion proteins

**Figure 1:**
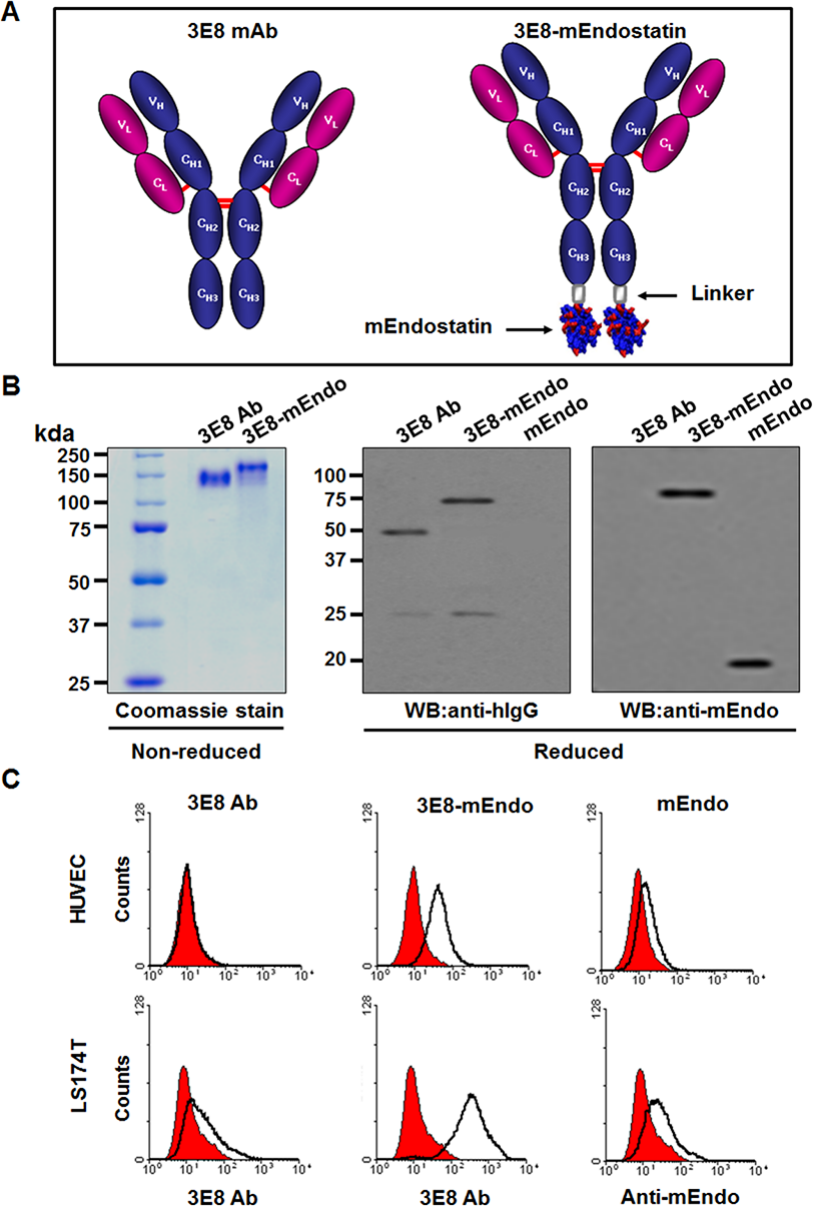
Purification and binding affinity of the 3E8-mEndo fusion protein **(A)** Schematic diagram of the anti-3E8 humanized antibody and the 3E8-mEndo fusion protein. **(B)** Expression of anti-3E8 antibody and 3E8-mEndo. Secreted 3E8 and 3E8-mEndo were analyzed under reducing and non-reducing conditions. **(C)** HUVECs and LS174T cells were treated with 3E8, 3E8-mEndo, and mEndo. The binding affinities of 3E8 and 3E8-mEndo were detected with the anti-3E8 antibody, and mEndo was detected by anti-mouse endostatin. Experiments were repeated three times. Isotype control, human IgG, filled with red.

We constructed and stably transfected an expression vector for the 3E8-mEndo fusion protein, comprising mouse endostatin (mEndo) fused to the C-terminal end of 3E8 antibody with a 17 amino acid-linker (Figure 1A), into dihydrofolate reductase-deficient CHO-DG44 cells. Due to the presence of an endogenous Igκ leader sequence, the expressed 3E8-mEndo fusion protein was secreted. Secreted 3E8 and 3E8-mEndo fusion proteins were purified from culture supernatants using protein A-sepharose affinity column chromatography. Purified 3E8 and 3E8-mEndo proteins were analyzed by SDS-PAGE under non-reducing or reducing conditions. Purified 3E8-mEndo migrated as a 190 kDa band under non-reducing conditions, indicating that it is composed of 3E8 (150 kDa) and two endostatin molecules (40 kDa) (Figure 1B). Western blot analyses using polyclonal anti-human IgG showed an approximately 25 kDa immunoreactive band corresponding to light chains of both 3E8 and 3E8-mEndo, as well as two bands with molecular masses of ~50 kDa and ~70 kDa, corresponding to heavy chain and heavy chain plus endostatin of 3E8 and 3E8-mEndo, respectively (Figure 1B). In contrast, immunoblotting with the anti-mEndostatin antibody only detects the 70 kDa polypeptide comprising heavy chain plus endostatin of the 3E8-mEndo fusion protein (Figure 1B).

Next, to determine whether the fusion of 3E8 and endostatin affects the binding ability to cells expressing TAG-72 antigen or endothelial cells, we analyzed the binding of 3E8, 3E8-mEndo, or mEndo to LS174T human colon carcinoma cells or HUVECs using flow cytometry. As shown in Figure 1C, 3E8 bound only to LS174T cells but did not bind to HUVECs, reflecting expression of TAG-72 in human colon carcinoma cells [12]. On the other hand, it has been reported that endostatin can bind to cell surface receptors including VEGFR-2 or basic fibroblast growth factor receptor-2 (FGFR-2) [13]. We determined the expression of those receptors in LS174T cells or HUVECs and observed that HUVECs express both VEGFR-2 and FGFR-2 but LS174T cells express only FGFR-2 (Supplementary Figure 1A and 1B). Consistent with the findings that mEndo binding was observed in LS174T cells and HUVECs, binding of the 3E8-mEndo fusion protein was significantly increased compared to 3E8 or mEndo in LS174T cells and HUVECs (Figure 1C). Because 3E8-mEndo fusion proteins are comprised of 3E8 and two molecules of endostatin, the increased binding of 3E8-mEndo can be explained by the sum of binding by each binding moiety of the fusion protein. Collectively, these results suggest that the 3E8-mEndo fusion protein can bind to carcinoma cells expressing TAG-72 antigen or endothelial cells at higher levels than 3E8 or endostatin.

### Inhibition of endothelial cell proliferation, migration, and tube formation by the 3E8-mEndo fusion protein

**Figure 2:**
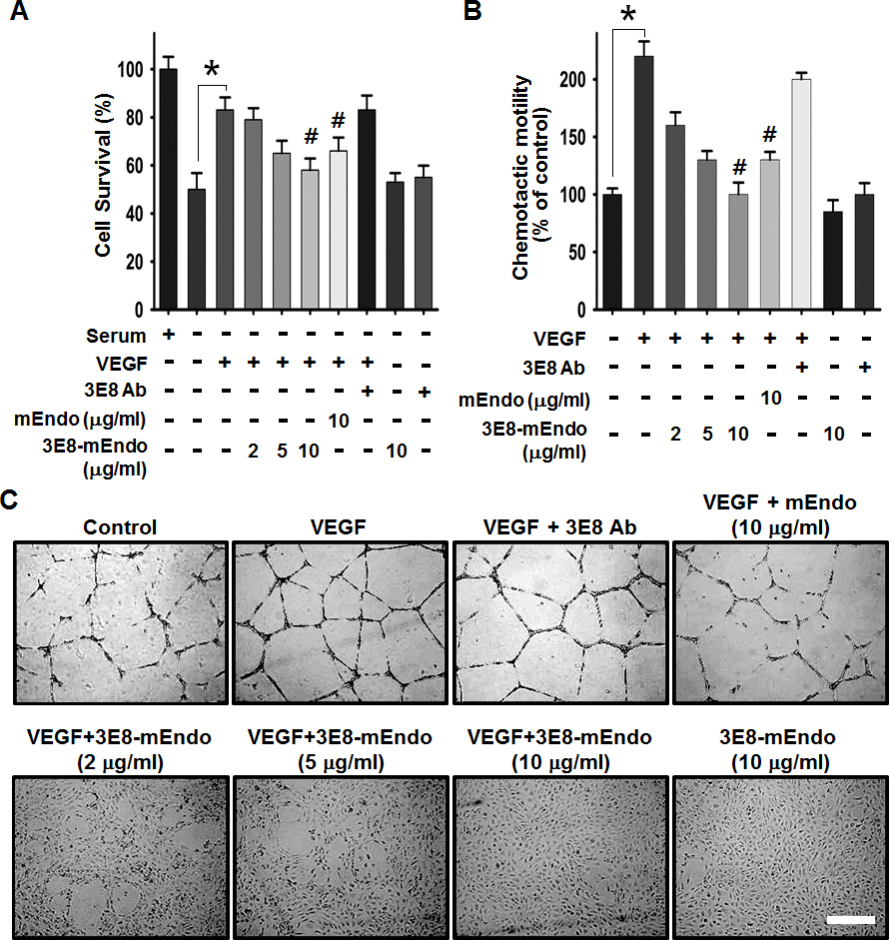
The effects of the 3E8-mEndo fusion protein on VEGF-induced endothelial cell proliferation, migration, and capillary-like tube formation **(A)** HUVECs were pretreated with 3E8 antibody, mEndo, and 3E8-mEndo for 30 min, and then stimulated with 10 ng/ml VEGF_165_. After incubation for 48 h, live cells were counted under a microscope. Each group was assayed in triplicate. **(B)** HUVECs were assayed for chemotaxis toward 10 ng/ml VEGF in the continued presence or absence of the fusion protein and mEndo. Cells that migrated to the bottom of transwell membranes were stained with hematoxylin and eosin and quantified under a microscope. Quantification of five independent assays is shown in the graphs (A and B). *p < 0.05; #*p* < 0.05 versus VEGF. Error bars indicate ± SEM. **(C)** HUVECs were resuspended in endothelial cell growth media and treated as indicated before plating onto Matrigel-coated plates. After incubation for 20 h, tube formation was observed using an inverted microscope. Each group was assayed in triplicate and experiments were repeated three times. Scale bar: 10 μm.

To determine whether the fusion of endostatin to 3E8 affects the anti-angiogenic activities of endostatin, we examined the effects of 3E8-mEndo on endothelial cell proliferation, migration, and capillary-like tube formation in response to VEGF. VEGF stimulation significantly induced HUVEC proliferation and migration (Figure 2A and 2B). However, pre-treating cells with mEndo or 3E8-mEndo significantly inhibited VEGF-induced proliferation and migration in HUVECs. The anti-angiogenic activity of the 3E8-mEndo fusion protein appears to be higher than mEndo because cells treated with 10 μg/ml of 3E8-mEndo more potently inhibited VEGF-induced proliferation and migration compared to cells treated with an equal concentration of mEndo. Additionally, the 3E8 antibody did not affect cell proliferation and migration in HUVECs in the presence or absence of VEGF. Moreover, cells treated with 3E8 or 3E8-mEndo without VEGF stimulation did not exhibit any toxicity. Next, we examined the effects of the 3E8-mEndo fusion protein on capillary-like tube formation *in vitro*. HUVECs were plated on growth factor-reduced Matrigel, and then pretreated with mEndo, 3E8, and 3E8-mEndo in the presence or absence of VEGF (10 ng/ml). Tube formation was determined by the presence of cells organized as closed polygons. mEndo treatment significantly reduced VEGF-induced tube formation, whereas the 3E8 antibody showed no significant effects. Interestingly, cells treated with 3E8-mEndo showed substantially inhibited VEGF-induced tube formation in a dose-dependent manner, which was dramatically higher than cells treated with mEndo.

### *Ex vivo* and *in vivo* anti-angiogenic activities of the 3E8-mEndo fusion protein

**Figure 3:**
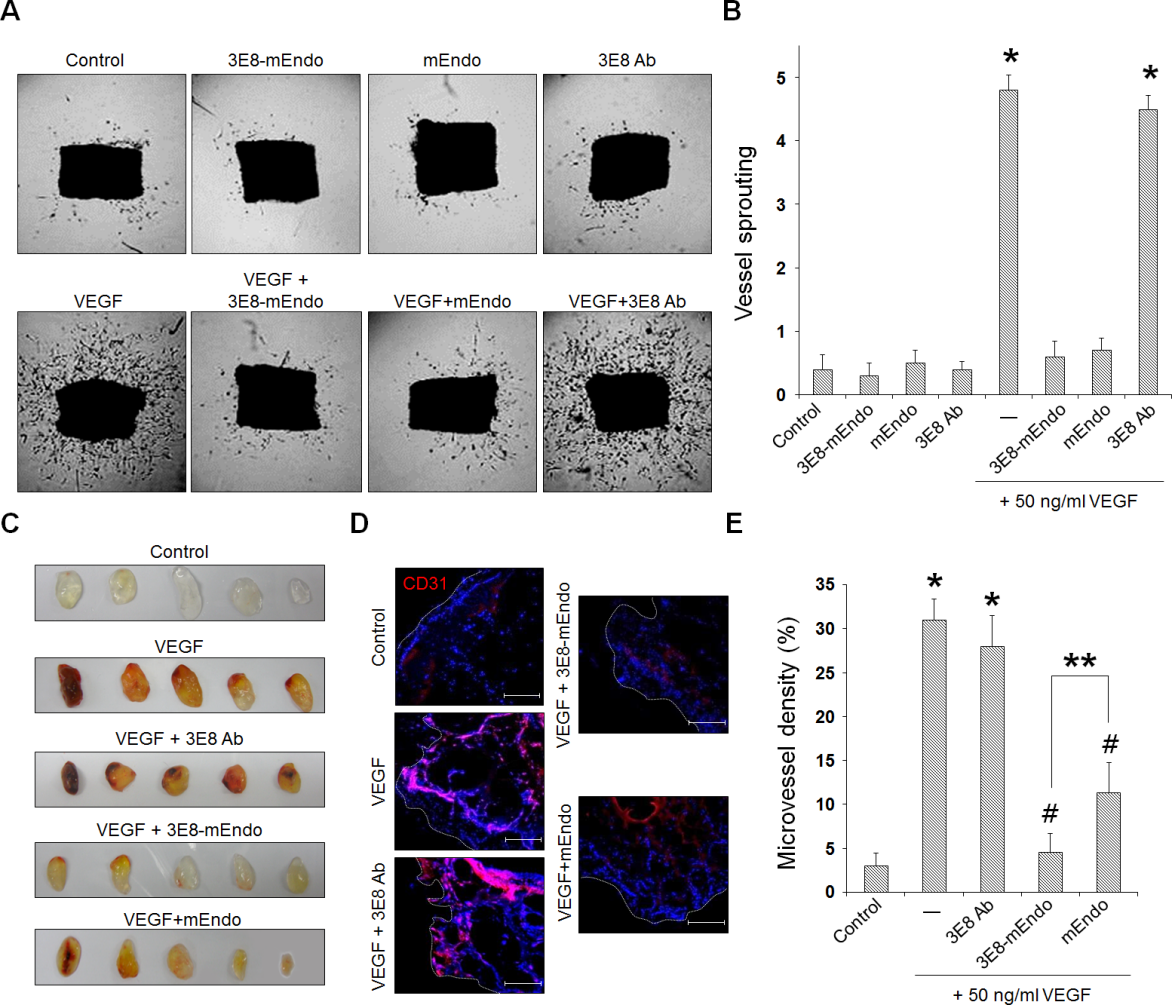
The 3E8-mEndo fusion protein has anti-angiogenic properties **(A)** Aortic segments were harvested from mice. Aortic segments in Matrigel were treated with 3E8-mEndo, 3E8 antibody, and mEndo in the presence or absence of VEGF_165_ (50 ng/ml) for 2 weeks (*n* = 5 per group). **(B)** Sprouting from aortic rings were classified as 0 (least positive) to 5 (most positive) as described in “Materials and Methods”. *p < 0.05 versus control; #*p* < 0.05 versus VEGF. Error bars indicate ± SEM. **(C)** C57BL/6 mice were injected with 0.6 ml of Matrigel containing VEGF with or without 3E8, 3E8-mEndo, and mEndo. After 7 days, Matrigel plugs were excised from the mice. **(D)** Plugs were stained for infiltrating endothelial cells using the anti-CD31 antibody. Red, CD31-positive; Blue, DAPI. **(E)** To quantify microvessel density (MVD), 5 random 0.42-mm^2^ fields in Matrigel plugs per each group was captured and CD31-positive vessel area was determined. Data are shown as mean ± SEM. *p < 0.05 versus control; #*p* < 0.05 versus VEGF only; **p < 0.05. Bars: 100 μm.

We further evaluated the anti-angiogenic activity of 3E8-mEndo using *ex vivo* and *in vivo* angiogenesis models. Mouse aortic rings were placed on growth factor-reduced Matrigel for vessel sprouting *ex vivo* assays. As shown in Figure 3A and 3B, vessel sprouting at the aortic rings was significantly increased by VEGF stimulation. However, treatment with mEndo or the 3E8-mEndo fusion protein significantly inhibited VEGF-induced vessel sprouting, whereas the 3E8 antibody did not show any inhibitory effects in the presence or absence of VEGF. In consistent with the finding that endostatin can bind to FGFR-2 as well as VEGFR-2 in endothelial cells, 3E8-mEndo also suppressed bFGF-induced arotic sprouting (Supplementary Figure 2). To further determine the inhibitory effects of 3E8-mEndo *in vivo*, we performed mouse Matrigel plug assays. Matrigel plugs in mice were excised and photographed. Matrigel plugs containing VEGF alone or in combination with the 3E8 antibody were red in color, indicating neovascularization (Figure 3C). In contrast, blood vessel formation was significantly decreased in Matrigel plugs containing mEndo or 3E8-mEndo in the presence of VEGF. Moreover, Matrigel plugs stained with the anti-CD31 antibody revealed more significant inhibition of neovascularization in plugs treated with 3E8-mEndo compared to mEndo (Figure 3D and 3E). Collectively, these results suggest that the anti-angiogenic activities of the 3E8-mEndo fusion protein were well preserved and perhaps more potent than mEndo both *in vitro* and *in vivo*.

### The 3E8-mEndo fusion protein inhibited VEGF signaling through the binding of VEGFR-2

**Figure 4:**
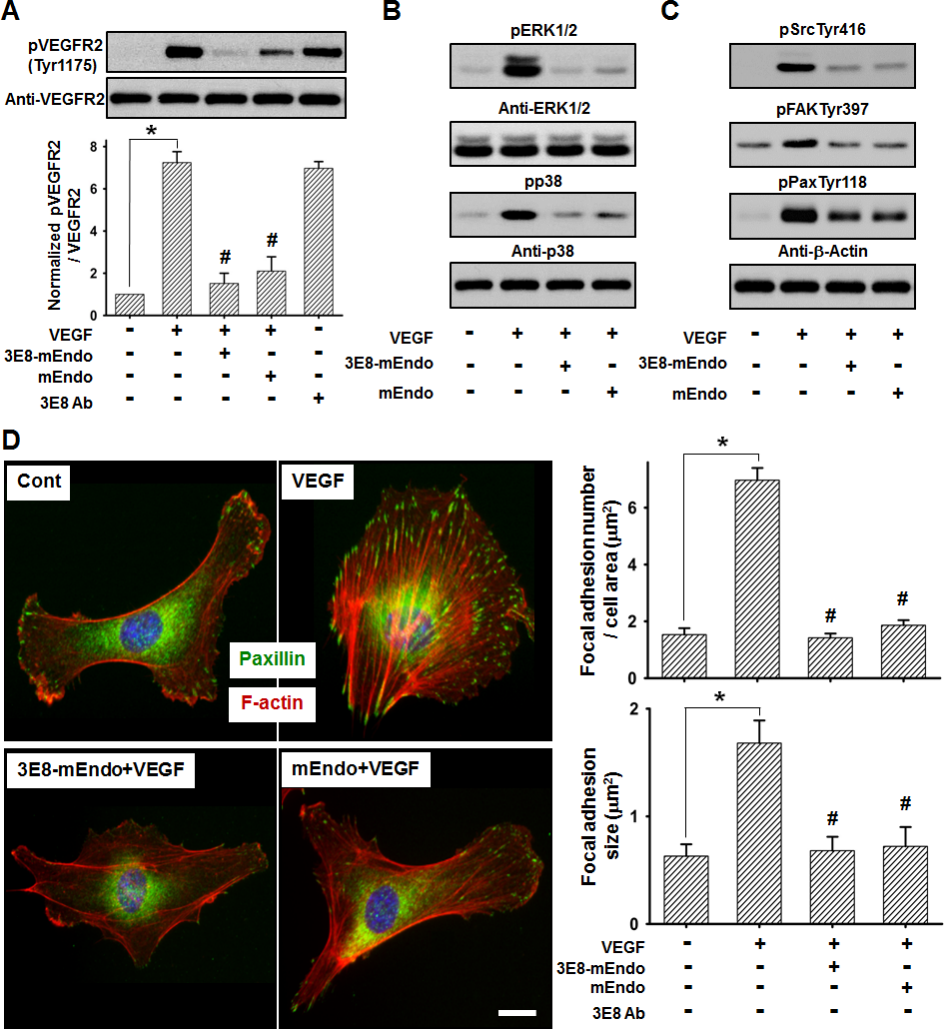
3E8-mEndo significantly inhibits VEGF receptor-2 activation **(A)** 3E8-mEndo and mEndo inhibit VEGF-induced VEGFR-2 phosphorylation. HUVECs were pretreated with 3E8-mEndo, mEndo, and 3E8 (10 μg/ml) for 30 min, and then stimulated with 10 ng/ml VEGF for 10 min. Cell lysates were collected and analyzed by western blotting using anti-pVEGFR2 at Tyr^1175^ and anti-VEGFR2 antibodies. Quantification of VEGFR2 phosphorylation from five independent experiments is shown in the graphs. *p < 0.05; #*p* < 0.05 versus VEGF. Error bars indicate ± SEM. **(B** and **C)** Pretreatment of HUVECs with 3E8-mEndo and mEndo (10 ug/ml) for 30 min, followed by stimulation with 10 ng/ml VEGF for 10 min. Cell lysates were collected and analyzed by western blotting. **(D)** The 3E8-mEndo fusion protein reduced VEGF-induced F-actin and focal adhesion formation. HUVECs were plated on coverslips coated with 20 μg/ml gelatin. After pretreatment with 3E8-mEndo and mEndo for 30 min, cells were stimulated with 10 ng/ml VEGF for 10 min. Immunostaining with the anti-mouse-paxillin antibody and TRITC-labeled phalloidin was performed on fixed cells. The size and number of focal adhesions were measured from the images (*n* = 15). *p < 0.05; #*p* < 0.05 versus VEGF. Error bars indicate ± SEM. Scale bar: 20 μm.

Based on the findings demonstrating that endostatin suppresses VEGF-induced downstream signaling that is involved in endothelial cell proliferation and migration [14, 15] by directly binding to VEGFR-2 [3], we determined the effects of 3E8-mEndo on VEGF-induced VEGFR-2 signaling pathways. Stimulation of cells with VEGF (10 ng/ml) significantly increased VEGFR-2 phosphorylation at Tyr^1175^. However, pretreatment of cells with 3E8-mEndo or mEndo significantly reduced VEGF-induced phosphorylation of VEGFR-2 at Tyr^1175^ (Figure 4A). However, cells treated with the 3E8 antibody alone were unaffected. Activation of ERK1/2 and p38 MAPK, as well as the Src-FAK signaling cascade, induced by VEGFR-2 phosphorylation plays an important role in endothelial cells proliferation and migration [3, 16]. The 3E8-mEndo fusion protein significantly blocked VEGF-induced ERK1/2 and p38 MAPK phosphorylation in HUVECs (Figure 4B). Moreover, treatment of HUVECs with 3E8-mEndo significantly inhibited VEGF-induced FAK, Src, and paxillin phosphorylation (Figure 4C). Immunofluorescent staining with F-actin and paxillin showed that actin stress fiber formation and the number and size of focal adhesion in VEGF-stimulated HUVECs were markedly increased. However, cells treated with 3E8-mEndo or mEndo prior to VEGF stimulation showed highly impaired actin stress fiber and focal adhesion formation (Figure 4D). Similarly, LS174T cells treated with 3E8-mEndo or mEndo showed significant inhibition of bFGF-induced FGFR-2 activation and the downstream signaling pathways including Akt and Src phosphorylation (Supplementary Figure 3). Collectively, these results indicate that 3E8-mEndo can suppress VEGFR-2 and FGFR-2 signaling pathways in HUVECs and LS174T cells.

### The 3E8-mEndo fusion protein is stable, and has improved tumor targeting and anti-tumor effects compared to commercial endostatin

**Figure 5:**
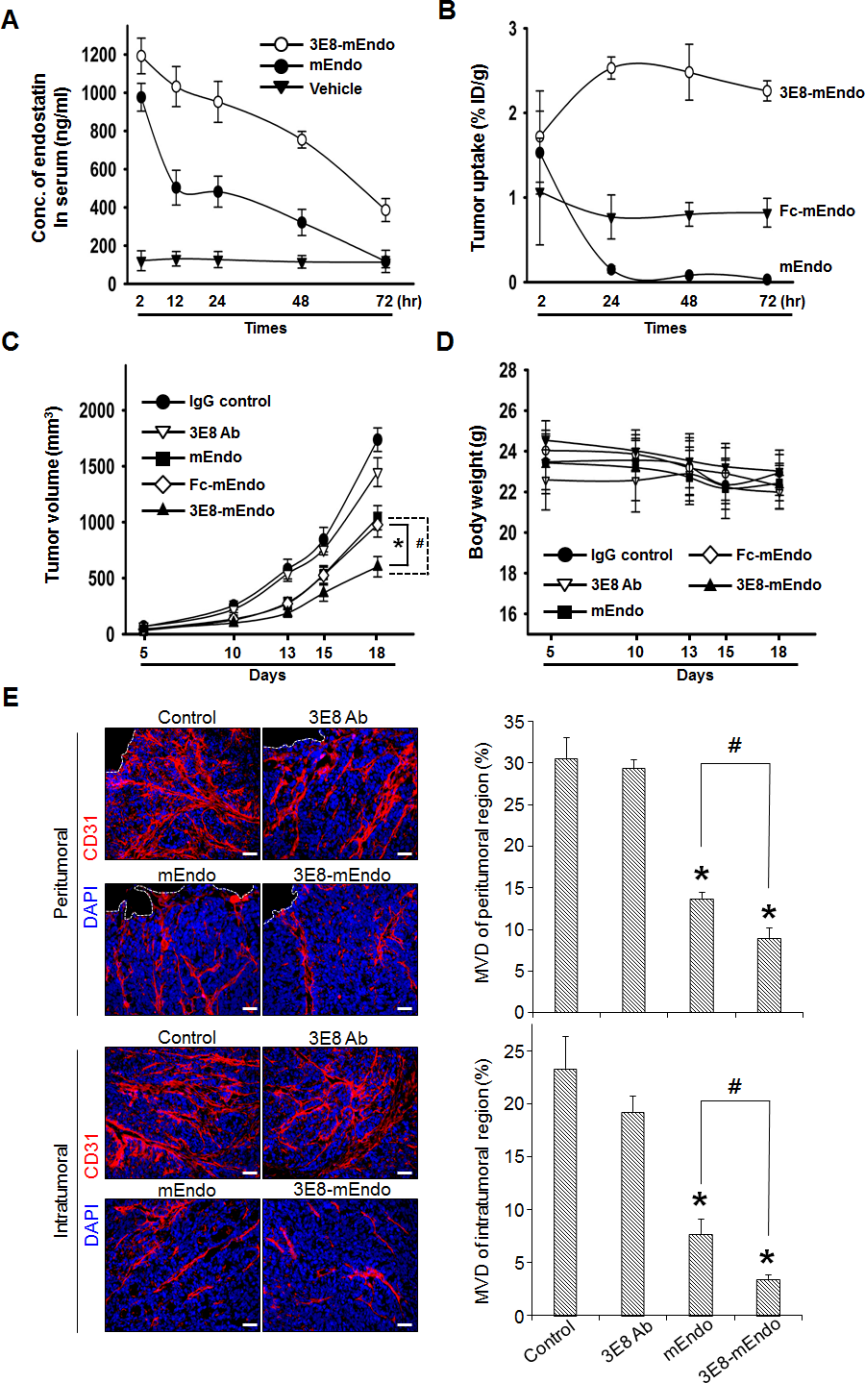
Anti-tumor effects of the 3E8-mEndo fusion protein **(A)** Pharmacokinetics of 3E8-mEndo and mEndo. mEndo (10 μg/0.1 ml) and 3E8-mEndo (47.5 mg, equimolar to 10 mg mouse endostatin) were injected via the tail vein of BALB/c mice (*n* = 5). At the indicated time points, serum samples were assayed using a mouse endostatin ELISA kit. **(B)** Biodistribution of ^125^I-labeled 3E8-mEndo, Fc-mEndo, and mEndo in athymic mice with LS174T tumor xenografts. ^125^I-3E8-mEndo, ^125^I-Fc-mEndo, and ^125^I-mEndo were injected intravenously into mice. At each time point, three mice were sacrificed. The percentages of the injected dose per gram (% ID/g) were determined in the tumor. **(C** and **D)** Human LS174T cells (5 × 10^6^) were implanted in the flank of athymic mice (*n* = 11), followed on day 6 by equimolar injections every other day (for a total of 8 times) of 3E8-mEndo (47.5 μg), 3E8 antibody (37.5 μg), Fc-mEndo (22.5 μg), and mEndo (10 μg). Tumor volumes were measured as described. *p < 0.05; #*p* < 0.05. Error bars indicate ± SEM. **(E)** Immunohistochemical staining of CD31-positive blood vessels in intra- and peritumoral regions of tumors from control or mice treated with 3E8, mEndo, or 3E8-mEndo, as indicated (left panels). To quantify microvessel density, 5 random 0.42-mm^2^ fields per each group were captured and CD31-positive area was determined (right panels). Error bars indicate ± SEM. *p < 0.01 versus control; #*p* < 0.05. Bars: 50 μm.

To characterize the pharmacokinetics of the 3E8-mEndo fusion protein and mEndo, mice were injected intravenously with equimolar amounts of 3E8-mEndo and mEndo. Whole blood was collected at the indicated time intervals. Serum concentrations of endostatin were measured using ELISA for mouse endostatin. mEndo was rapidly eliminated from the serum in mice, 12 h after injection. In contrast, similar serum levels of 3E8-mEndo were maintained 72 h post-injection (Figure 5A).

Lee *et al*. [17] reported that Fc fusion to endostatin significantly improves the *in vivo* half-life and efficacy of endostatin. To evaluate the tumor-targeting capability of 3E8-mEndo independent from the effects of its prolonged *in vivo* half-life, we first measured the localization of 3E8-mEndo, Fc-mEndo, and mEndo to the antigenic tumor mass site. Athymic mice were injected subcutaneously with LS174T human colon adenocarcinoma cells. When the implanted tumors became palpable, ^125^I-3E8-mEndo, ^125^I-Fc-mEndo, or ^125^I-mEndo was injected into the tumor-bearing mice. Tumors were collected 2, 24, 48, and 72 h post-injection. The localization of each protein in the tumor was calculated as the percentage of the injected dose/g of tumor tissue (Figure 5B). ^125^I-mEndo levels in tumors rapidly decreased 24 h after the dosing, whereas ^125^I-Fc-mEndo levels were maintained at steady-state levels over the experimental period. However, significantly higher localization was observed in the tumor tissues of mice given ^125^I-3E8-mEndo proteins compared to those treated with ^125^I-Fc-mEndo or ^125^I-mEndo. ^125^I-3E8-mEndo levels peaked at 24 h and were maintained until 72 h post-dosing. These data indicate that 3E8-mEndo fusion proteins are localized to tumor tissues by their ability to bind TAG-72 antigens in the tumor cells, rather than by their prolonged *in vivo* half-life.

To determine whether the prolonged serum half-life of 3E8-mEndo and its localization into tumor tissues translates to improved anti-tumor efficacy, we examined the anti-tumor activity of the 3E8-mEndo fusion protein against human colon cancer LS174T xenografts in athymic mice. Equimolar doses of each protein were injected every other day for 2.5 weeks. Compared to IgG-treated control mice, 3E8-treated mice exhibited no significant inhibition of tumor growth, whereas mice treated with mEndo or Fc-mEndo showed significant inhibition of tumor growth (Figure 5C) and more do so in mice treated with 3E8-mEndo fusion protein. Moreover, no significant general toxicities were observed in control or treated mice, as determined by loss of body weight (Figure 5D). To delineate whether the suppression of tumor growth may be resulted from the decreased tumor angiogenesis, we analyzed microvessel density (MVD) in tumors from control and mice treated with 3E8, mEndo, or 3E8-mEndo. A significant correlation between the suppression of tumor growth and the decreased tumor angiogenesis was observed. As shown in Figure 5E, MVD in tumors from 3E8-mEndo- or mEndo-treated mice was significantly reduced in both peri- and intratumoral regions and the reduction in MVD was more significant in tumors from mice treated with 3E8-mEndo than mEndo, whereas treatment with 3E8 did not decrease the MVD. Collectively, these results strongly suggest that fusion of the 3E8 antibody to endostatin is a useful strategy for the treatment of tumors expressing the TAG-72 antigen.

## DISCUSSION

Clinical trials using human endostatin has failed because they did not show a significant anti-tumor efficacy in cancer patients [18–21]. This failure can be explained, in part, by its short *in vivo* half-life. Additionally, peak kinetics resulting from bolus administration of recombinant endostatin may be insufficient to maintain prolonged therapeutic levels in the tumor mass. Many recombinant protein therapeutics with molecular masses ≤ 50 kDa (including 20 kDa endostatin) are rapidly cleared from circulation by glomerular filtration and degradation in the renal tubules. Thus, these proteins have short *in vivo* half-lives of a few minutes to a few hours. In the present study, we generated a fusion protein of murine endostatin to a humanized antibody against TAG-72 antigen. The resultant fusion protein, designated 3E8-mEndo, is a secreted protein of ~190 kDa. Importantly, the half-life of 3E8-mEndo was significantly increased compared to murine endostatin. Similarly, Fc- or anti-HER2 antibody fusion to endostatin showed prolonged half-lives in circulation. For example, the elimination half-life (t_1/2_) of the anti-HER2-endostatin fusion protein (40.2 ± 2.7 h) is ~10 times longer than that of endostatin (3.75 ± 2.18 h) in normal mice [22]. Antibody fusion did not adversely affect the anti-angiogenic activities of endostatin. In fact, the anti-angiogenic potency of 3E8-mEndo appears to be enhanced, resulting in part from the two-fold increased avidity of endostatin in the fusion protein. Consistently, endothelial cells treated with dimeric or trimeric endostatin significantly inhibited tube formation compared to cells treated with endostatin monomers [23].

Due to the prolonged half-life in circulation, the Fc-Endostatin fusion protein was reported to have ~100 times higher anti-tumor efficacy than endostatin in an animal model [17]. However, the local concentration of therapeutic proteins in the tumor appears to be more important than the level in the systemic circulation because the primary targets of endostatin are the highly proliferating tumor-associated endothelial cells. In this respect, we selected the anti-TAG-72 antibody as a fusion partner due to its specific binding ability to the TAG-72 antigen in tumors, including colon carcinoma. The anti-TAG-72 antibody has long been used as a radioimmunotherapeutic agent in the treatment of ovarian cancer, indicating that TAG-72 is a type of tumor-specific marker [24, 25]. Adenovirus carrying fused CC49, a humanized anti-TAG-72 monoclonal antibody, can transfer a target gene selectively to ovarian carcinoma cells without Coxsackie-adenovirus receptor and significantly decreased adenovirus-mediated toxicity was observed [26]. Moreover, several nanoparticles conjugated with the anti-TAG-72 antibody, as well as a fluorophore-labeled anti-TAG-72 antibody have also been used as targeting agents for radioimmunotherapy and tumor imaging [27–30]. Because TAG-72 is specifically expressed in colon carcinoma tissues, we attempted to target colon carcinoma using 3E8-mEndo in an effort to improve the anti-tumor efficacy of this fusion protein. Interestingly, 3E8-mEndo proteins were highly localized to the implanted colon carcinoma tissues. This improved retention in the tumor mass appears not to result from an extended half-life in the circulation because 3E8-mEndo was more markedly retained than Fc-mEndo, which has a similar *in vivo* half-life. Moreover, tumor growth suppression in mice treated with 3E8-mEndo was significantly higher compared to Fc-mEndo or mEndo, and 3E8 antibodies did not show any significant activity.

Angiogenesis is considered as an independent prognostic marker for metastatic colon carcinoma patients [31, 32]. VEGF-induced signaling through VEGFR-2 is a main axis that controls tumor angiogenesis. Warren *et al*. [33] demonstrated that VEGF and VEGFR-2 are highly expressed in both primary colorectal carcinomas and their liver metastases, and play a critical role in tumor growth and metastasis. VEGFR-2 inhibition using a VEGFR-2-specific inhibitor (SU5416) or multiple tyrosine kinase inhibitor (SU6668) revealed significant inhibition of colorectal cancer growth and metastasis with significantly decreased microvessel density [34]. These data suggest that targeting VEGF signaling is a powerful approach to inhibit the growth and metastasis of human colon carcinoma. Therefore, the finding that endostatin suppresses VEGF signaling in endothelial cells by directly interfering with VEGFR-2 activation, thus inhibiting angiogenesis *in vitro* and *in vivo*, may have clinical implications.

Although we clearly demonstrate that the 3E8-mEndo fusion protein has improved anti-angiogenic activities, an extended half-life in circulation, higher localization in the tumor mass, and significantly enhanced anti-tumor activities compared endostatin, the following issues must be considered before clinical application of this fusion protein in anti-cancer therapeutics. Although it was not shown in the present study, endostatin has been reported to have a biphasic U-shaped curve of anti-tumor activities. Therefore, well-designed translational experiments are required to optimize its anti-tumor efficacy and other clinical parameters, including the optimal dose and dosing schedules. In addition, the anti-tumor efficacy of anti-angiogenic agents, including endostatin, could be significantly increased if used in combination with conventional chemotherapeutic agents. Accordingly, various combinations of 3E8-mEndo and other therapeutic modalities should also be tested. Contrary to the initial expectations, prolonged anti-VEGF therapy with bevacizumab, a humanized monoclonal VEGF neutralizing antibody, is reported to be associated several clinical toxicities [35]. Moreover, tumor cells can escape anti-VEGF therapy by producing other angiogenic factors. Therefore, because angiogenesis inhibitors would be administered for long periods of time, possible long-term safety concerns should also be assessed.

In conclusion, our data clearly demonstrate that fusing the 3E8 antibody to endostatin is a promising approach to extend the *in vivo* half-life of endostatin and to improve its anti-angiogenic and anti-tumor efficacy. Due to the powerful tumor-targeting ability of the anti-TAG-72 antibody, this strategy can be expanded to other anti-angiogenic proteins for the treatment of various carcinomas expressing TAG-72.

## METERIALS AND METHODS

### Cell culture and reagents

Human umbilical vein endothelial cells (HUVECs) were isolated from human umbilical cord veins by collagenase treatment as described previously [36]. Cells from passages 2–7 were subsequently used in experiments. HUVECs were grown in M199 media (Welgene, Daegu, Korea) supplemented with 20% fetal bovine serum (FBS), 100 units/ml penicillin, 100 μg/ml streptomycin, 3 ng/ml of basic fibroblast growth factor (Millipore, Billerica, MA, USA), and 5 units/ml heparin (Sigma-Aldrich, St. Louis, MO, USA) at 37°C under a humidified 95% and 5% (v/v) mixture of air and CO_2_. LS174T human colorectal carcinoma cells and dihydrofolate reductase (DHFR)-deficient Chinese hamster ovary DG44 (CHO-DG44) cells were grown in Dulbecco's modified eagle media (DMEM) or minimum essential media (MEM)-α (Gibco, Grand Island, NY, USA), respectively, supplemented with 100 units/ml penicillin, 100 μg/ml streptomycin, and 10% FBS or 10% dialyzed FBS (for CHO-DG44 cell). VEGF_165_ and mouse endostatin (mEndo) were purchased from R&D Systems (Minneapolis, MN, USA). Matrigel was purchased from BD Biosciences (San Jose, CA, USA). Anti-human IgG and anti-mouse endostatin antibodies were purchased from Sigma. Anti-CD31, phospho-VEGF receptor 2 (Tyr^1175^), pSrcTyr^416^, pERK1/2, pFAKTyr^397^, and anti-phospho-paxillin Tyr^118^ were purchased from Cell Signaling (Beverly, MA, USA).

### Construction, expression, and purification of endostatin fusion proteins

The cDNA encoding mouse endostatin, corresponding to the carboxyl terminal 183 amino acids of mouse collagen XVIII (Genbank Accession no. A1326391), was synthesized by reverse transcription-polymerase chain reaction (RT-PCR) by using the following primers: the forward primer (5′-ATAAGAATGCGGCCGCTTC TGGTGCTGGAGCTTCAGGCATGGTGCATCCGGTTC GGGATCTGGAAGCCATACTCATCAGGACTTTCAGC CAGTG-3′), which contained DNA sequences corresponding to the *Not*I restriction enzyme sequence, a linker with 17 amino acids, and nine N-terminal amino acids of mouse endostatin; the reverse primer (5′-ATAAGAATGCGGCCGCCTATTTGGA GAAAGAGG TCATGAAGCTATTCTC-3′), which contained DNA sequences corresponding to the C-terminal 10 amino acids of mouse endostatin, a stop codon, and the *Not*I restriction enzyme sequence. After the digestion with *Not*I, the PCR product was subcloned into the re-digested pdCMV-dhfr-3E8 vector to construct the plasmid p3E8-mEndo, which expresses a fusion protein of mouse endostatin fused to the C-terminal end of the heavy chain constant domain (C_H_3) of the 3E8 anti-TAG-72 humanized antibody through a 17 amino acid-linker peptide. DNA sequences for the fusion protein of mouse endostatin fused to the C-terminus of the Fc domain (C_H_2–C_H_3) of 3E8 (Fc-mEndo) were PCR amplified using primers (primer 1, 5′-GACGAATTCACTCTAACCATGGAA-3′; primer 2, 5′-AGTTTTGTCGACCTGGGAGAGGACACCTG TA GT-3′; primer 3, 5′-CTCTCCCAGGTCGACAAAACTC ACACATGCCCACCGTGC-3′; primer 4, 5′-CCGCTC GACCTACTTGGAGGCAGTCATGAAGCTG-3′). Primer 1 and 4 contained *Eco*RI and *Xho*I restriction enzyme sequences. The final fragment was digested with *Eco*RI/*Xho*I and subcloned into the pJK-dhfr vector to construct expression plasmid, Fc-mEndo.

For the stable expression of the 3E8-mEndo fusion protein, the p3E8-mEndo expression vector was transfected into CHO-DG44 cells using Lipofectamine 2000 according to the manufacturer's recommendations (Invitrogen, Carlsbad, CA, USA). After selection in MEM-α containing G418 in 96-well plates, resistant cell clones were screened for the production of the assembled fusion protein using an indirect ELISA and subjected to step-wise methotrexate adaptation as described previously [37]. Fc-mEndo was transiently expressed in HEK 293T cells using Lipofectamine 2000 (Invitrogen) and collected from culture supernatants.

For production and purification of fusion proteins, the CHO cell line that stably expressed the antibody was grown in serum-free media (CHO-SFM II; Invitrogen). Culture supernatants were subjected to affinity chromatography on protein A-sepharose columns (Amersham Biosciences) as described previously [38]. Columns were eluted with 0.1 M citric acid (pH 3.5) and immediately neutralized with 1 M Tris (pH 8.8). After overnight dialysis with PBS (pH 7.4) at 4°C, fusion protein concentrations were determined using ELISA. The integrity and purity of the purified antibody were determined by SDS-PAGE.

### Flow cytometry analyses

LS174T cells and HUVECs were detached with cell dissociation buffer (Gibco), and treated with 3E8 (37.5 μg/ml, equimolar to 10 μg mEndo), 3E8-mEndo (47.5 μg/ml, equimolar to 10 μg mEndo), and/or mEndo (10 μg/ml) for 30 min at 37°C. Cells were then blocked with 1% bovine serum albumin (BSA) in PBS for 30 min at room temperature. Cells treated with 3E8 or 3E8-mEndo were incubated with the anti-3E8 antibody (10 μg/ml) or human IgG (isotype control; 10 μg/ml) at 4°C for 1 h in PBS containing 1% BSA. Cells treated with mEndo were incubated with the anti-mEndo antibody for 1 h at 4°C. After incubation, cells were washed three times with 1% BSA in PBS and stained with fluorescein isothiocyanate (FITC)-conjugated anti-human IgG for 30 min at 4°C. After washing twice with PBS, cells were stained with propidium iodide (PI) to discriminate dead cells and live cells. Cells that negatively stained with PI were gated using a FACS caliber (BD Immunocytometry System, San Jose, CA, USA) and used for subsequent antibody binding experiments.

### Endothelial cell proliferation and migration assays

Endothelial cell proliferation assays were performed as previously described [39]. Briefly, HUVECs (2 × 10^5^ cells/well) were seeded onto gelatin-coated 6-well plates for 24 h, washed with M199, and the culture media were replaced with M199 containing 1% FBS for 6 h. Cells were then treated with 3E8 (10 μg/ml), mEndo (10 μg/ml), or 3E8-mEndo (2, 5, or 10 μg/ml) proteins for 30 min, and incubated for 48 h in the presence or absence of 10 ng/ml VEGF_165_, as indicated. Cell viability was analyzed using Trypan blue dye exclusion tests. Only viable cells were counted.

Transwell chemotaxis assays were performed as described previously [39]. Briefly, transwell chamber membranes (6.5 mm diameter, 8 μM pore size; Corning Costar, Corning, NY, USA) were coated with 10 μg/ml gelatin. For chemotaxis assays, 20 ng/ml VEGF in M199 media (1% FBS) were added to the lower chamber. HUVECs (1 × 10^6^ cells/ml) were suspended in M199 containing 1% FBS. Various concentrations of the 3E8-mEndo fusion protein, 10 μg/ml of 3E8 antibody, and/or 10 μg/ml of mouse endostatin were added to the cells for 30 min at room temperature before seeding. Cell suspensions (100 μl) were added to the upper chamber. After incubating for 4 h, cells remaining in the upper chamber were removed using a cotton swab. Cells that migrated to the lower surface of the membrane were fixed and stained with hematoxylin and eosin. Chemotaxis was quantified by counting cells that migrated to the lower side of the membrane using light microscopy. Ten random fields were counted for each assay.

### Matrigel tube formation assays

Tube formation was assayed as previously described [40]. In brief, 250 μl growth factor-reduced Matrigel (BD Biosciences, San Jose, CA, USA) was pipetted into 24-well tissue culture plates and polymerized for 30 min at 37°C. HUVECs were harvested, and 1.5 × 10^5^ cells were resuspended in M199 media containing 1% FBS. Cells were pretreated with the indicated concentrations of 3E8-mEndo, mEndo, or 3E8 for 30 min, plated onto the Matrigel layer, and 10 ng/ml VEGF was added. After incubation for 20 h at 37°C, vascular tube-like structures were quantified using an inverted microscope.

### Aortic ring assays

Aortas were harvested from C57BL/6 mice (aged 7 weeks) and sectioned into several pieces (aortic rings). Each well of 48-well plates was coated with 120 μl Matrigel. The aortic rings were placed on Matrigel-coated wells, and covered with an additional 50 μl of Matrigel. VEGF (50 ng/ml) with or without 3E8-mEndo, mEndo, or 3E8, was added to the wells in a final volume of 200 μl endothelial basal media (EBM; Lonza). Plates were incubated at 37°C, and media were changed every 2 days for 2 weeks. Angiogenic sprouting from aortic rings was examined in five rings per group (*n* = 5). Each aortic ring was photographed, and sprouting was quantified by counting the number of vascular sprouts that directly originated from the aorta. Photographed images were divided into 5 regions, and sprouts in each region were scored from 0 (least positive) to 5 (most positive) in a double-blind manner. Each data point was assayed five times.

### *In vivo* matrigel plug assays

Matrigel plug assays were performed as previously described [40]. Briefly, C57BL/6 mice were subcutaneously injected with 0.6 ml of Matrigel containing 200 ng VEGF and 10 units heparin, with or without the 3E8-mEndo fusion protein (47.5 μg, equimolar to 10 μg mouse endostatin), mEndo (10 μg), or 3E8 (37.5 μg, equimolar to 10 μg mEndo). The injected Matrigel rapidly formed a single, solid gel plug. After 7 days, the skin of the mouse was pulled back to expose the Matrigel plug, which remained intact. To identify infiltrating endothelial cells, immunohistochemistry was performed using the anti-CD31 antibody (BD Biosciences).

### Immunoblotting and immunofluorescence assays

Cell lysates from HUVECs were analyzed by SDS-PAGE and transferred to PVDF membranes. Blocked membranes were incubated with the indicated primary and secondary antibodies. Immunoreactive bands were visualized using chemiluminescence, followed by exposure to X-ray film. Bands were quantified using ImageJ software.

For focal adhesion and F-actin formation, cells were attached to glass coverslips coated with 20 μg/ml gelatin. After starvation for 6 h in M199 media containing 1% FBS, cells were pretreated with 3E8-mEndo or mEndo, and stimulated with 10 ng VEGF. Cells were washed once in PBS, fixed for 10 min in 3.7% formaldehyde, permeabilized for 20 min at room temperature with 0.2% Triton X-100, washed in PBS, and blocked for 30 min at room temperature in 1% bovine serum albumin in PBS. After incubation for 1 h with the paxillin antibody, cells were washed in PBS and incubated for 1 h with FITC-conjugated anti-mouse antibodies and TRITC-labeled phalloidin (Sigma) in PBS. Cells were washed with PBS, stained with DAPI for nuclei staining, and coverslips were mounted on the slide. Images were captured with an Olympus DP30BW digital camera and processed using the Metamorph 7.1 program (Universal Imaging, Media, PA, USA).

### Pharmacokinetics of 3E8-mEndo fusion proteins

To monitor the levels of 3E8-mEndo fusion proteins in serum, BALB/c mice (*n* = 5, aged 5–6 weeks) were intravenously (i.v.) injected with either 3E8-mEndo (47.5 μg, equimolar to 10 μg mouse endostatin) or mouse endostatin (10 μg). Blood samples were serially collected at indicated time intervals by retroorbital puncture with non-heparinized capillary tubes. Samples were placed at room temperature for 30 min, and serum was collected after centrifugation at 1,000 × g for 15 min. Concentrations were determined using mouse endostatin ELISA kits (Cusabio, Wuhan, Hubei, China) following the manufacturer's protocol.

### Biodistribution studies

Athymic mice (BABL/c-*nu/nu*; aged 5–6 weeks) were obtained from RaonBio (Yongin, GyeonGi-do, Korea). Human colon adenocarcinoma cells LS174T (5 × 10^6^) were subcutaneously injected into the right thigh. After 12 days, ^125^I-3E8-mEndo, ^125^I-Fc-mEndo, or ^125^I-mEndo (20 μg/740 KBq) were injected into the tail vein of athymic mice bearing LS174T tumors. For each time point, three mice were sacrificed to collect tumors. Radioactivity was measured using a γ-scintillation counter. The percentage of the injected dose/g of tumor was calculated.

### *In vivo* tumor growth assays

To evaluate anti-tumor activity of 3E8-mEndo fusion proteins *in vivo*, human LS174T colon cancer cells (5 × 10^6^) were subcutaneously (s.c.) implanted in the flank of athymic mice (BABL/c-*nu/nu*; aged 5–6 weeks; *n* = 11 per group). Beginning on day 5, mice were injected i.v. with equimolar amounts of purified 3E8-mEndo (47.5 μg), 3E8 antibody (37.5 μg), Fc-mEndo (22.5 μg), and mEndo (10 μg) as indicated. This treatment was repeated every other day for 8–9 doses as indicated. Mice were weighed and tumors monitored three times per week in 2 diameters using digital calipers. Tumor volumes were determined using a^2^ × *b* × 0.5, where a is the shortest diameter and b is the longest diameter.

### Statistical analyses

Data are expressed as the mean ± standard error of the mean (SEM). Differences between groups were analyzed using Student's *t*-tests. *p* < 0.05 was considered statistically significant. Data are representative of at least three independent experiments.

## SUPPLEMENTARY FIGURES



## References

[1] Folkman J (2006). Angiogenesis. Annual review of medicine.

[2] O’Reilly MS, Boehm T, Shing Y, Fukai N, Vasios G, Lane WS, Flynn E, Birkhead JR, Olsen BR, Folkman J (1997). Endostatin: an endogenous inhibitor of angiogenesis and tumor growth. Cell.

[3] Kim YM, Hwang S, Pyun BJ, Kim TY, Lee ST, Gho YS, Kwon YG (2002). Endostatin blocks vascular endothelial growth factor-mediated signaling via direct interaction with KDR/Flk-1. The Journal of biological chemistry.

[4] Wickstrom SA, Alitalo K, Keski-Oja J (2002). Endostatin associates with integrin alpha5beta1 and caveolin-1, and activates Src via a tyrosyl phosphatase-dependent pathway in human endothelial cells. Cancer research.

[5] Hanai J, Gloy J, Karumanchi SA, Kale S, Tang J, Hu G, Chan B, Ramchandran R, Jha V, Sukhatme VP, Sokol S (2002). Endostatin is a potential inhibitor of Wnt signaling. The Journal of cell biology.

[6] Hanai J, Dhanabal M, Karumanchi SA, Albanese C, Waterman M, Chan B, Ramchandran R, Pestell R, Sukhatme VP (2002). Endostatin causes G1 arrest of endothelial cells through inhibition of cyclin D1. The Journal of biological chemistry.

[7] Yu HK, Lee HJ, Ahn JH, Lim IH, Moon JH, Yoon Y, Yi LS, Kim SJ, Kim JS (2013). Immunoglobulin Fc domain fusion to apolipoprotein(a) kringle V significantly prolongs plasma half-life without affecting its anti-angiogenic activity. Protein engineering, design & selection: PEDS.

[8] Carter P (2001). Improving the efficacy of antibody-based cancer therapies. Nature reviews Cancer.

[9] Schrama D, Reisfeld RA, Becker JC (2006). Antibody targeted drugs as cancer therapeutics. Nature reviews Drug discovery.

[10] Johnson VG, Schlom J, Paterson AJ, Bennett J, Magnani JL, Colcher D (1986). Analysis of a human tumor-associated glycoprotein (TAG-72) identified by monoclonal antibody B72.3. Cancer research.

[11] Thor A, Ohuchi N, Szpak CA, Johnston WW, Schlom J (1986). Distribution of oncofetal antigen tumor-associated glycoprotein-72 defined by monoclonal antibody B72.3. Cancer research.

[12] Yoon SO, Lee TS, Kim SJ, Jang MH, Kang YJ, Park JH, Kim KS, Lee HS, Ryu CJ, Gonzales NR, Kashmiri SV, Lim SM, Choi CW, Hong HJ (2006). Construction, affinity maturation, and biological characterization of an anti-tumor-associated glycoprotein-72 humanized antibody. The Journal of biological chemistry.

[13] Blackhall FH, Merry CL, Lyon M, Jayson GC, Folkman J, Javaherian K, Gallagher JT (2003). Binding of endostatin to endothelial heparan sulphate shows a differential requirement for specific sulphates. The Biochemical journal.

[14] Veikkola T, Alitalo K (1999). VEGFs, receptors and angiogenesis. Seminars in cancer biology.

[15] Zachary I (2001). Signaling mechanisms mediating vascular protective actions of vascular endothelial growth factor. American journal of physiology Cell physiology.

[16] Koch S, Tugues S, Li X, Gualandi L, Claesson-Welsh L (2011). Signal transduction by vascular endothelial growth factor receptors. The Biochemical journal.

[17] Lee TY, Tjin Tham Sjin RM, Movahedi S, Ahmed B, Pravda EA, Lo KM, Gillies SD, Folkman J, Javaherian K (2008). Linking antibody Fc domain to endostatin significantly improves endostatin half-life and efficacy. Clinical cancer research: an official journal of the American Association for Cancer Research.

[18] Eder JP, Supko JG, Clark JW, Puchalski TA, Garcia-Carbonero R, Ryan DP, Shulman LN, Proper J, Kirvan M, Rattner B, Connors S, Keogan MT, Janicek MJ, Fogler WE, Schnipper L, Kinchla N (2002). Phase I clinical trial of recombinant human endostatin administered as a short intravenous infusion repeated daily. Journal of clinical oncology: official journal of the American Society of Clinical Oncology.

[19] Hansma AH, Broxterman HJ, van der Horst I, Yuana Y, Boven E, Giaccone G, Pinedo HM, Hoekman K (2005). Recombinant human endostatin administered as a 28-day continuous intravenous infusion, followed by daily subcutaneous injections: a phase I and pharmacokinetic study in patients with advanced cancer. Annals of oncology: official journal of the European Society for Medical Oncology/ESMO.

[20] Herbst RS, Hess KR, Tran HT, Tseng JE, Mullani NA, Charnsangavej C, Madden T, Davis DW, McConkey DJ, O’Reilly MS, Ellis LM, Pluda J, Hong WK, Abbruzzese JL (2002). Phase I study of recombinant human endostatin in patients with advanced solid tumors. Journal of clinical oncology: official journal of the American Society of Clinical Oncology.

[21] Kulke MH, Bergsland EK, Ryan DP, Enzinger PC, Lynch TJ, Zhu AX, Meyerhardt JA, Heymach JV, Fogler WE, Sidor C, Michelini A, Kinsella K, Venook AP, Fuchs CS (2006). Phase II study of recombinant human endostatin in patients with advanced neuroendocrine tumors. Journal of clinical oncology: official journal of the American Society of Clinical Oncology.

[22] Shin SU, Cho HM, Merchan J, Zhang J, Kovacs K, Jing Y, Ramakrishnan S, Rosenblatt JD (2011). Targeted delivery of an antibody-mutant human endostatin fusion protein results in enhanced antitumor efficacy. Molecular cancer therapeutics.

[23] Kuo CJ, LaMontagne KR, Garcia-Cardena G, Ackley BD, Kalman D, Park S, Christofferson R, Kamihara J, Ding YH, Lo KM, Gillies S, Folkman J, Mulligan RC, Javaherian K (2001). Oligomerization-dependent regulation of motility and morphogenesis by the collagen XVIII NC1/endostatin domain. The Journal of cell biology.

[24] Alvarez RD, Partridge EE, Khazaeli MB, Plott G, Austin M, Kilgore L, Russell CD, Liu T, Grizzle WE, Schlom J, LoBuglio AF, Meredith RF (1997). Intraperitoneal radioimmunotherapy of ovarian cancer with 177Lu-CC49: a phase I/II study. Gynecologic oncology.

[25] Meredith RF, Partridge EE, Alvarez RD, Khazaeli MB, Plott G, Russell CD, Wheeler RH, Liu T, Grizzle WE, Schlom J, LoBuglio AF (1996). Intraperitoneal radioimmunotherapy of ovarian cancer with lutetium-177-CC49. Journal of nuclear medicine: official publication, Society of Nuclear Medicine.

[26] Kelly FJ, Miller CR, Buchsbaum DJ, Gomez-Navarro J, Barnes MN, Alvarez RD, Curiel DT (2000). Selectivity of TAG-72-targeted adenovirus gene transfer to primary ovarian carcinoma cells versus autologous mesothelial cells *in vitro*. Clinical cancer research: an official journal of the American Association for Cancer Research.

[27] Milenic DE, Brady ED, Garmestani K, Albert PS, Abdulla A, Brechbiel MW (2010). Improved efficacy of alpha-particle-targeted radiation therapy: dual targeting of human epidermal growth factor receptor-2 and tumor-associated glycoprotein 72. Cancer.

[28] Sharifzadeh Z, Rahbarizadeh F, Shokrgozar MA, Ahmadvand D, Mahboudi F, Jamnani FR, Moghimi SM (2013). Genetically engineered T cells bearing chimeric nanoconstructed receptors harboring TAG-72-specific camelid single domain antibodies as targeting agents. Cancer letters.

[29] Xu JS, Huang J, Qin R, Hinkle GH, Povoski SP, Martin EW, Xu RX (2010). Synthesizing and binding dual-mode poly (lactic-co-glycolic acid) (PLGA) nanobubbles for cancer targeting and imaging. Biomaterials.

[30] Zou P, Xu S, Povoski SP, Wang A, Johnson MA, Martin EW, Subramaniam V, Xu R, Sun D (2009). Near-infrared fluorescence labeled anti-TAG-72 monoclonal antibodies for tumor imaging in colorectal cancer xenograft mice. Molecular pharmaceutics.

[31] Takahashi Y, Tucker SL, Kitadai Y, Koura AN, Bucana CD, Cleary KR, Ellis LM (1997). Vessel counts and expression of vascular endothelial growth factor as prognostic factors in node-negative colon cancer. Arch Surg.

[32] Takebayashi Y, Aklyama S, Yamada K, Akiba S, Aikou T (1996). Angiogenesis as an unfavorable prognostic factor in human colorectal carcinoma. Cancer.

[33] Warren RS, Yuan H, Matli MR, Gillett NA, Ferrara N (1995). Regulation by vascular endothelial growth factor of human colon cancer tumorigenesis in a mouse model of experimental liver metastasis. The Journal of clinical investigation.

[34] Ellis LM, Takahashi Y, Liu W, Shaheen RM (2000). Vascular endothelial growth factor in human colon cancer: biology and therapeutic implications. The oncologist.

[35] Ferrara N, Hillan KJ, Gerber HP, Novotny W (2004). Discovery and development of bevacizumab, an anti-VEGF antibody for treating cancer. Nature reviews Drug discovery.

[36] Jaffe EA, Nachman RL, Becker CG, Minick CR (1973). Culture of human endothelial cells derived from umbilical veins. Identification by morphologic and immunologic criteria. The Journal of clinical investigation.

[37] Kim SJ, Jang MH, Stapleton JT, Yoon SO, Kim KS, Jeon ES, Hong HJ (2004). Neutralizing human monoclonal antibodies to hepatitis A virus recovered by phage display. Virology.

[38] Ryu CJ, Padlan EA, Jin BR, Yoo OJ, Hong HJ (1996). A humanized antibody with specificity for hepatitis B surface antigen. Human antibodies and hybridomas.

[39] Min JK, Cho YL, Choi JH, Kim Y, Kim JH, Yu YS, Rho J, Mochizuki N, Kim YM, Oh GT, Kwon YG (2007). Receptor activator of nuclear factor (NF)-kappaB ligand (RANKL) increases vascular permeability: impaired permeability and angiogenesis in eNOS-deficient mice. Blood.

[40] Kim SJ, Lee Y, Kim NY, Hwang Y, Hwang B, Min JK, Koh SS (2013). Pancreatic adenocarcinoma upregulated factor, a novel endothelial activator, promotes angiogenesis and vascular permeability. Oncogene.

