# Supplementary material for: Extension of the in vivo half-life of endostatin and its improved anti-tumor activities upon fusion to a humanized antibody against tumor-associated glycoprotein 72 in a mouse model of human colorectal carcinoma
Source: Oncotarget. 2015 Jan 22;6(9):7182–94. doi: 10.18632/oncotarget.3121 (PMC4466677; doi:10.18632/oncotarget.3121)
Supplement: Supplementary file 1 [file oncotarget-06-7182-s001.pdf]

SUPPLEMENTARY FIGURES

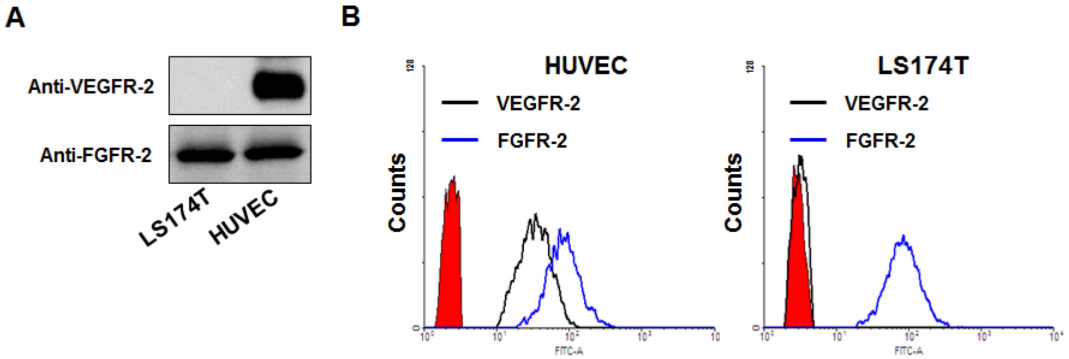

Supplementary Figure 1: Expression of VEGFR-2 and FGFR-2 in the LS174T cells and human umbilical vein endothelial cells (HUVECs). (A) Cells were lysed and subjected to Western blotting with anti-VEGFR-2 and anti-FGFR-2 antibodies. **(B)** The expression levels of VEGFR-2 (black lines) and FGFR-2 (blue lines) at the cell surface were analyzed by flow cytometry. Human IgG was used as a negative control (filled in red).

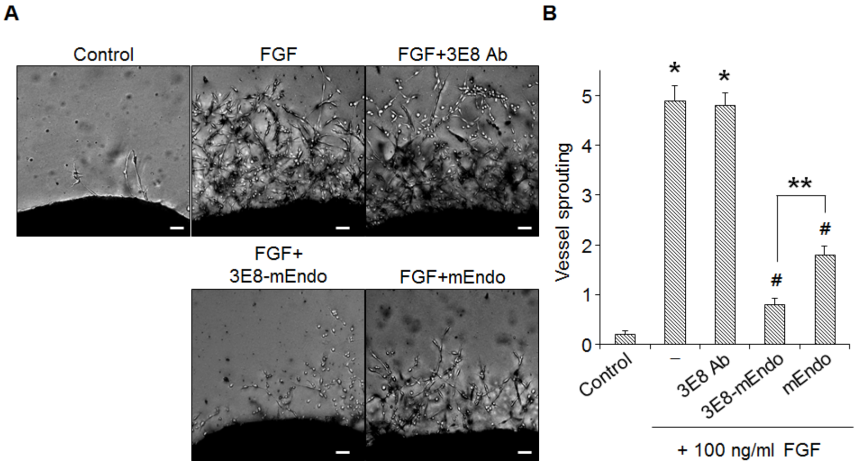

Supplementary Figure 2: Inhibition of bFGF-induced vessel sprouting by 3E8-mEndo fusion proteins in the aortic ring assay. (A) Aortic segments were harvested from mice. Aortic segments in Matrigel were treated with 3E8-mEndo, 3E8 antibody, or mEndo in the presence of bFGF (100 ng/ml) for 1 weeks (n = 5 per group). **(B)** Sprouting from aortic rings were classified as 0 (least positive) to 5 (most positive) as described in “Materials and Methods”. *$p < 0.01$ versus control; #$p < 0.05$ versus FGF; **$p < 0.05$. Error bars indicate ± SEM.

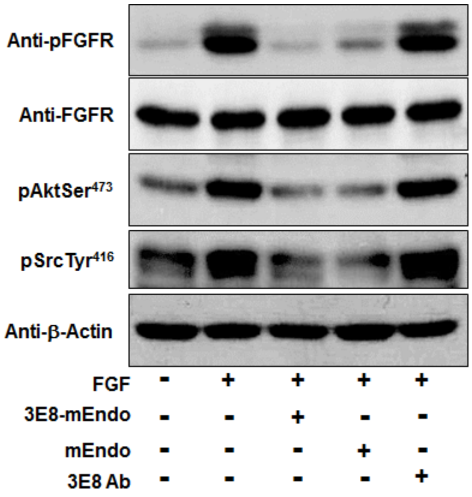

Supplementary Figure 3: Inhibition of bFGF-induced FGFR-2 receptor activation and its downstream signaling by 3E8-mEndo. Cells were pretreated with 3E8-mEndo, mEndo, or 3E8 (10 μg/ml) for 30 min, and then stimulated with 50 ng/ml FGF for 5 min. Cell lysates were collected and analyzed by Western blotting using indicated antibodies.
